# Ten principles of good interdisciplinary team work

**DOI:** 10.1186/1478-4491-11-19

**Published:** 2013-05-10

**Authors:** Susan A Nancarrow, Andrew Booth, Steven Ariss, Tony Smith, Pam Enderby, Alison Roots

**Affiliations:** 1 Southern Cross University, Military Road, East Lismore, 2480, Australia; 2 University of Sheffield, Regent Court, 30 Regent Street, Sheffield, South Yorkshire, S1 4DA, UK; 3 Sheffield Hallam University, Collegiate Crescent, Sheffield, South Yorkshire, S10 2BP, UK; 4 University of Victoria, 3800 Finnerty Road, Victoria, BC, V8P5C2, Canada

**Keywords:** Interdisciplinary team work, Competencies, Intermediate care, Transitional care, Allied health, Systematic review, Evidence synthesis, Qualitative research

## Abstract

**Background:**

Interdisciplinary team work is increasingly prevalent, supported by policies and practices that bring care closer to the patient and challenge traditional professional boundaries. To date, there has been a great deal of emphasis on the processes of team work, and in some cases, outcomes.

**Method:**

This study draws on two sources of knowledge to identify the attributes of a good interdisciplinary team; a published systematic review of the literature on interdisciplinary team work, and the perceptions of over 253 staff from 11 community rehabilitation and intermediate care teams in the UK. These data sources were merged using qualitative content analysis to arrive at a framework that identifies characteristics and proposes ten competencies that support effective interdisciplinary team work.

**Results:**

Ten characteristics underpinning effective interdisciplinary team work were identified: positive leadership and management attributes; communication strategies and structures; personal rewards, training and development; appropriate resources and procedures; appropriate skill mix; supportive team climate; individual characteristics that support interdisciplinary team work; clarity of vision; quality and outcomes of care; and respecting and understanding roles.

**Conclusions:**

We propose competency statements that an effective interdisciplinary team functioning at a high level should demonstrate.

## Background

Interdisciplinary team work is a complex process in which different types of staff work together to share expertise, knowledge, and skills to impact on patient care. Despite increasing emphasis on interdisciplinary team work over the past decade, in particular the growth of interdisciplinary education [1], there is little evidence as to the most effective way of delivering interdisciplinary team work [2]. This difficulty is compounded by the multifactorial nature of team work, which comprises the skill mix, setting of care, service organisation, individual relationships and management structures.

Most existing research explores the impact of one or a few of these aspects, rather than examining the relationships among several of these components on a range of staff and patient outcomes. Similarly, interventions designed to improve interdisciplinary team work tend to focus on specifics of team work activities such as: sharing of patient files [3], case-conferencing approaches [4,5], or meeting style or frequency [6-10]. To date, there is not a systematic framework around which these activities, or characteristics, of interdisciplinary working can be structured.

### Terminology

A wide range of terms are used to describe collaborative working arrangements between professionals [11]. Terms such as interdisciplinary, interprofessional, multiprofessional, and multidisciplinary are often used interchangeably in the literature to refer to both different types of teams and different processes within them [12]. They are also often used in conjunction with the term team work. However, there are some consistent distinctions that are useful to understand. The terms inter/multi-professional are generally narrower than the terms inter/multi-disciplinary [13-16] and refer to teams consisting exclusively of professionals from different professions or disciplines, or at least to the relationships between professionals in teams that may also include other non-professional staff. The terms inter/multi-disciplinary are broader and include all members of healthcare teams, professional and non-professional. Other authors have suggested use of the prefixes multi-, inter- and trans- to reflect differing intensities of integration [17].

The focus of this paper is on inter/multi-disciplinary teams: the research, interventions, and data-gathering activities underpinning the study included all members of the respective healthcare teams. The term “interdisciplinary team” is used as a generic term of reference for these healthcare teams which included a range of health service workers, both professionals and non-professionals, with the majority being from professional groups. However, where authors have used the terms inter/multi/trans-professional or inter/multi-disciplinary the authors’ original terms will be used.

### Interdisciplinary team work

Previous research has investigated the fundamental concepts and features associated with team work. A concept analysis [18] to explore the basic understanding of team work in the healthcare context drew on both healthcare and literature from other disciplines such as human resource management, organisational behaviour, and education, and proposed the following definition for team work in the health care context:

“A dynamic process involving two or more health professionals with complementary backgrounds and skills, sharing common health goals and exercising concerted physical and mental effort in assessing, planning, or evaluating patient care. This is accomplished through interdependent collaboration, open communication and shared decision-making. This in turn generates value-added patient, organisational and staff outcomes.” (p.238).

This definition may be more optimistic and aspirational than realistic as it makes several assumptions about the characteristics that a team will possess. Enderby [19] identified these characteristics to include a definable membership, group consciousness, shared vision, corporate sense of purpose, clear interdependence and interaction, and co-ordinated action.

Xyrichis and Ream’s [18] literature analysis concludes that the outcomes from team work could be experienced at three levels (healthcare professionals, patients, and healthcare organizations) and that these outcomes have an impact on staff satisfaction, quality of care, control of costs, well-being and retention. Molyneux [20] identified three indicators for positive team work: personal qualities and commitment of staff, communication within the team, and the opportunity to develop creative working methods within the team. Further literature reviews [11] have identified the importance of two themes on interprofessional team work, team structure and team processes within which specific categories emerged: team premises, team size and composition, organizational support, team meetings, clear goals and objectives, and audit processes.

Collaboration is acknowledged as an important component of team processes. A concept analysis undertaken by Henneman *et al*. [21] identified that collaboration “requires competence, confidence and commitment on the part of all parties. Respect and trust, both for oneself and others, is key to collaboration. As such, patience, nurturance and time are required to build a relationship so that collaboration can occur” (p.108). Identified factors that contribute to successful collaboration were: joint venture, cooperative endeavor, willing participation, shared planning and decision-making, team approach, contribution of expertise, shared responsibility, non-hierarchical relationships and shared power based on knowledge and expertise [21]. However, further reviews [22] have found that the reality of shared planning and decision-making, and shared power is very different from the ideal. Given the context of interprofessional teams, members will automatically come from different professions, therefore in practice “shared decision-making” is likely to conflate individual team members making decisions within their own scope of practice with the ideal of all team members sharing in all decision-making processes, or in other words, “appropriate” decision making. Shared power and leadership may also be a challenge when complex traditional hierarchical relationships, particularly those involving medical practitioners, play a larger role and impact either implicitly or explicitly on team processes [23,24]. McCallin [23] suggests that shared leadership occurs only in smaller teams privileged with being free to choose all team members.

When considering the characteristics important for interprofessional team work within the context of organisational development, McCray [25] points out that little attention appears to have been paid to the actual processes of interprofessional practice within organisational strategy, local workforce development planning, and individual continuing professional development.

### Necessity of interdisciplinary team work

The need for interdisciplinary team work is increasing as a result of a number of factors including:

(1) an aging population with frail older people and larger numbers of patients with more complex needs associated with chronic diseases;

(2) the increasing complexity of skills and knowledge required to provide comprehensive care to patients;

(3) increasing specialization within health professions and a corresponding fragmentation of disciplinary knowledge resulting in no-one health care professional being able to meet all the complex needs of their patients;

(4) the current emphasis in many countries’ policy documents on multi-professional team work and development of shared learning; and,

(5) the pursuit of continuity of care within the move towards continuous quality improvement [26].

Workforce re-structuring to meet these needs requires that interdisciplinary teams must integrate changing organisational values with new modes of service delivery [13]. While these changes impact across healthcare as a whole, there are certain sectors where these organisational challenges have encountered more widespread debate, in particular primary care, rehabilitation, and care of the elderly. Of these, primary care is perceived to have the least likely level of success with interdisciplinary team work. Indeed, some commentators suggest that an interdisciplinary culture may only be possible as new generations of healthcare professionals enter the workforce [27].

Despite the increasing focus on interdisciplinary team work over the past two decades, there is still no clear synthesis of the “essence” of what makes a good interdisciplinary team and a lack of empirical research to define what such a team might look like. Similarly, there is a lack of data identifying the processes of interdisciplinary team work and linking these with outcomes. Studies tend to focus on processes or outcomes, but rarely both; or explore components of what defines an interdisciplinary team, without providing a clear guide on the attributes of good interdisciplinary team practice.

This paper draws on a published systematic review of the literature [28], combined with empirical data derived from interdisciplinary teams involved in the delivery of community rehabilitation and intermediate care services (CRAICs), to develop a set of competencies around effective interdisciplinary team practice. The research was contextualised in CRAICs.

CRAICs in England are community-based services frequently offering care for the elderly aimed at preventing admissions and facilitating earlier discharge from acute care. They exemplify the practice of interdisciplinary team work. Typically, CRAICs employ at least four different staff types, including nurses, physiotherapists and occupational therapists [29]. They often exhibit high levels of joint working and role sharing, and employ a large proportion of support workers who, when used appropriately, have been shown to facilitate interdisciplinary practice in this setting [29]. However, previous research by our team found a great deal of variety in the way that teams work together, and their levels of effectiveness as teams [30]. In response, we developed an Interdisciplinary Management Tool (IMT) which was implemented iteratively, using an action research approach with 11 teams to explore the impact of the tool on those teams and their patient outcomes [31].

## Methods

This research formed part of a much larger project designed to develop, implement and evaluate an intervention to enhance interdisciplinary team work [28] through the development of an IMT [32]. The IMT is a structured change management approach which marries published research evidence relating to interdisciplinary team work with the tacit knowledge of the particular team to develop a tailored approach to optimize their interdisciplinary team work [33]. Development of the tool involved three systematic reviews, interactions with team members using an action research methodology, and capturing extensive, detailed qualitative and quantitative feedback from teams and service users.

The findings presented in this paper draw on a systematic review of the literature relating to the components of interdisciplinary team work and the qualitative data derived from the implementation of the IMT. This latter component of the study included the exploration of team members’ perceptions of the important components underpinning interdisciplinary team work. Themes from these two perspectives were then examined for areas of agreement and dissonance to arrive at a set of competencies for good interdisciplinary team work.

### Systematic review

The systematic review, reported and published in full in the main study report [31], first considered quantitative studies; in particular randomised controlled trials (RCTs) published and unpublished between 1994 and 2009, that evaluated the process and outcomes of different interprofessional staffing models. Reference lists associated with the identified reports and articles were also searched for additional studies. Results were limited to English language articles in recognition of the importance of cultural factors in team work, and issues relating to differences in terminology (for example, multi-, inter-, trans- and cross- disciplinary working). A total of 153 studies, including 11 systematic reviews or meta-analysis, were reviewed and analysed; however, only 101 were usable based on the supporting level of contextual detail. Data on team effectiveness was extracted along with details on team processes, coordination, and leadership; all elements identified as important in the earlier concept analysis of the interdisciplinary team [18].

This initial review confirmed that a lack of contextual detail, both in trials in general [33], and interdisciplinary studies in particular [34], makes it difficult to isolate the “ingredients” of effective interdisciplinary team work. Specifically, the research team identified a lack of “thickness” in the detail on context, team roles, and processes from the review of the RCTs. In the absence of mixed-method studies, suggested as a priority for future research by a recent review [34], the team designed a supplementary review strategy. This strategy examined findings from qualitative research on interprofessional team processes, independent from the RCTs. Inclusion criteria for the supplementary review were studies between 2000 and 2010 involving an interprofessional team in CRAICs which included data focused on team processes. This complementary review identified 20 studies to supplement previous findings. The findings of the separate evidence bases from qualitative and quantitative studies were brought together and isolated to a data extraction table. Themes were identified using a constant comparative method [35] and then each study was coded appropriately. The constant comparative method involves the incorporation, collation and comparison of newly collected data with existing or previous data collected from earlier studies. Thematic synthesis was used to look for common patterns across studies [36].

### Team perspectives

Eleven CRAICs, including 253 staff were recruited to participate in an action research study, which examined the impact of implementing the IMT on service provision and outcomes for patients and staff. NHS ethics approval was obtained on 11 September 2008 (08/H1004/124). All participating team members provided written consent for their involvement in this research.

The IMT intervention was implemented through a series of semi-structured workshops with the support of a trained facilitator. These workshops included an initial, full day “Service Evaluation Conference” to ascertain each team’s values, needs, and priorities, and then a series of half day “Team Learning Sets” designed to allow for reflective evaluation of their team practice. The activities undertaken included the identification of issues and priority actions that each team wanted to pursue and exploration of what they considered to be “characteristics of a good team”. The workshop outcomes were detailed in reports and action plans that guided the implementation of their proposed changes. These reports and plans provided the basis of the data for the team perspectives. The data were entered into NVivo version 8.0 and coded thematically to explore the characteristics of a good team.

## Results

### Results from the thematic synthesis of the literature

Through the use of the constant comparative method, the thematic synthesis of the literature identified sixteen analytical themes with up to 12 descriptive characteristics in each theme. The identified themes and their characteristics are presented in Table 1.

**Table 1:** Results from the thematic synthesis of the literature

**Themes**	**Characteristics**		
Climate	• Interprofessional atmosphere	• Team culture	• Trust
	• Valued contributions	• Nurturing consensus	• Participative safety
			• Personal qualities
Communication	• Formal/Informal structures	• Completion/Reading care plans	• Use of shared case notes
	• Intra-team communication		• Regular case conferences
Individual characteristics	• Knowledge/experience	• Interpersonal team relationships	• Common goals
	• Interpersonal skills	• Listening skills	• Different opinions/perceptions
	• Personal characteristics	• Understanding own role/others roles	• Exploring/Acceptance role overlap
Interdependence	• Mutual support	• Willingness to share	• Professional synergy
	• Reciprocity within team	• Team relationships	
Leadership	• Role of physicians	•Need for chairperson role	
Learning	• Action based learning	• Nurturing a learning culture	• Training within clinical teams
	• Interprofessional learning		
Patient focus	• Patient centeredness	• Outcomes focus	• Team care planning and discussion
	• Holistic care	• Timely interventions	• Impacts of reduced contact time
Perceptions	• Differing perceptions of own role, others roles, team work		
Power	• Equality of relationships	• Hierarchical/traditional role of medicine	• Assertiveness/confidence
	• Power/Status	• Reluctance to voice opinions	• Scapegoat (Victimization)
Problem solving/decision-making	• Proactive approach	• Physician role	
	• Creativity		
Professional commitment	• Professional identity	• Professional jargon	• Tensions/rivalry
	• Role expectations	• Knowledge/skills	• Jealousy
Roles	• Autonomy	• Role enactment	• Role boundaries/delineation/decision making
	• Role modeling	• Role clarity	
Skills	• Core professional competencies, skills, tasks	• Sharing of knowledge/information/skills	• Differing levels of skill acquisition
Structures	• Organizational factors	• Goal planning	• Time
	• Team building	• Common location	• Team meetings/case conferences
Team characteristics	• Capacity	• Size	• Accessibility after hours
	• Dynamics/Balance	• Membership	
Values	• Philosophy	• Shared goals/objectives	• Practice context
	• Staff commitment		• Positive attitude

These themes highlight the defining characteristics of interdisciplinary team work. They recognize the important role that leadership plays for the complex communication and coordination necessary among different groups of professionals and non-professionals. They also demonstrate the need for both flexibility and clarity of roles when the bodies of knowledge of distinctive professional groups are shared, protected, and preserved.

### Results from the data on team perspectives

Two activities undertaken as part of the IMT workshops provided data representing the teams’ perceptions of the important components of interdisciplinary team work. These activities were (1) the identification of characteristics associated with ‘a good team’ and, (2) the challenges chosen by the teams as issues on which they considered focusing their action plans.

The characteristics associated with a good team identified by the 11 teams were grouped into13 themes (Table 2). In addition the 584 identified issues or challenges were grouped to 11 broad topics (Table 3). These two thematic analyses were then combined with the themes identified from the systematic review to form a single theoretical framework to define the characteristics of a good interdisciplinary team.

**Table 2:** Characteristics of a “good team” as identified by team members

	
1. Good communication	Communication primarily referred to intra-team communication and included team members feeling as though they could listen as well as speak out within a team context; and the ability to discuss and resolve difficulties within the team. It was suggested that being part of a large team hinders good communication by limiting the “two-way” communication, and that some peoples' views do not travel “upwards”.
2. Respecting/understanding roles	Importance of respecting and understanding the roles of other team members; that the limitations and boundaries of each role were well understood; and to have an understanding of how the roles have the potential to impact on patients. Practitioners should also be aware of how their own role fits within the team, and differs from that of other team members, and that roles and responsibilities are made explicit.
3. Appropriate skill mix	Skill mix refers to the mix and breadth of staff, personalities, individual attributes, professions and experience. Teams value diversity, and clearly need input from a range of staff who bring complementary experience and attributes to the team. Teams also felt that it was important to have the full complement of staff.
4. Quality and outcomes of care	Ensuring the quality and outcomes of care was identified as an important component of a good team and includes several reflective mechanisms both within and external to the team. Teams emphasized the importance both to have systems for capturing their effectiveness (such as measuring patient outcomes); and to meet their targets. This included suggestions that teams are able to reflect; accept criticism and act on it; have defined outcomes; follow-up patients; provide feedback to other services (for example, on appropriateness of referrals and timeliness and appropriateness of information provided); and celebrate their own successes; and clinicians keeping their skills up to date.
5. Appropriate team processes and resources	This theme includes access to sufficient physical resources (office space, parking, computers); privacy to make confidential phone calls; appropriate and efficient systems and procedures, including induction processes, policies, and paperwork that serves the need of the service whilst avoiding duplication. Workload management, having enough time to do the job, and time management were highlighted by several teams. Finally, the pathway for patients, and the integration of the team with wider services was seen as an important procedural issue.
6. Clear vision	Participants identified the need for a clear vision, role and purpose of the team. This was both to steer the direction of the team, but also required so that teams could establish appropriate referral criteria into the team.
7. Flexibility (of the team and the individuals within it)	The need for flexibility was identified as an individual attribute “ability to cover each other’s roles, but knowing your boundaries”. Individuals also need to be flexible to respond to the constantly changing service environment and patient needs (for instance, flexibility of working hours). Flexibility of the service was also identified, for instance, flexibility in referral criteria.
8. Leadership and management	All teams identified the importance of good leadership, and the characteristics of a good leader are explored elsewhere.
9. Team culture: camaraderie and team support/relationships	The importance of team culture was the largest theme, with 66 items within this theme. Trust, mutual respect, reliability, commitment and support were the most commonly raised themes. But team culture included the importance of informal relationships, camaraderie, fun, and friendship between colleagues.
10. Training and development opportunities	Opportunities for gaining new knowledge, sharing knowledge, continuing professional development, and education.
11. External image of the service	The importance of the external image of the service was raised by half of the teams and included the physical presentation of the staff (that is, whether or not they wear uniforms); the external image portrayed to outside agencies through their external points of contact (for instance phone systems that do not work properly); the external marketing of the service, which is important for managing referrals and the workload of the team.
12. Personal attributes	Several personal attributes were identified as being important to having an excellent team. These included approachability, appropriate delegation, being able to compromise, confidentiality, decisiveness, empathy, good organisation skills, initiative; knowing ones strengths and weaknesses; open to learning; acquiring, demonstrating and sharing new skills and knowledge, patience, personal responsibility, protective, reflexive practice, tolerance
13. Individual rewards and opportunity	Participants identified the importance of the individual returns on team work, which included good financial rewards; opportunities for career development; autonomy; challenge within the role and the opportunity to think outside the box.

**Table 3:** Challenges to interdisciplinary team work identified by teams

**Code name**	**Code description**	**Inclusion**	**Exclusion**
Clarity of vision, uncertainty and changes to service	The extent to which values are shared by team members including goals and objectives of the team and definitions of the service.	Including uncertainty at strategic level, external pressure to change and ways of managing change.	Excluding issues around clear delineation of individual roles and better understanding of others' roles/professions (5).
			Excluding individual goals (6).
Communication and relationships-external	Communication and relationships with external organizations/services and senior management.	Knowledge of other services. Including external factors which affect the team and the influence of the team on external services and organizations.	Excluding issues related to change and uncertainty (3).
Communication and relationships-internal	General team relationship and communication issues.	Including team integration, clear knowledge of others' roles and meetings.	Excluding joint working, sharing skills & knowledge and reflective practices (8).
CPD, rotation and career progression	Activities aimed at professional development: training, knowledge, skills, rotation, secondment and opportunities for promotion and progression.	Including individual goals and personal issues, for example, anxiety and self-worth.	
Facilities, resources, procedures and administration	Issues relating to facilities, resources and working practices and procedures.		Excluding capacity/team size, workload & time-management (11).
Joint-working	Activities related to staff members working together and observing each others’ work.	Including joint visits and assessments and shadowing opportunities.	
Management, leadership, decision-making and autonomy	Explicit mentions of managers and management or leaders and leadership and euphemisms (for example. higher level), especially regarding decision making and coordination.	Includes processes of decision making within the team including decisions being made by superiors and having autonomy to make own decisions	Excluding issues covered by other codes for example, working procedures (7), staffing levels (11), clarity of goals (3), communication (4 and 5), de-briefing .procedures (13) and so on.
Morale and motivation	Issues reported to positively or negatively affect the morale of team members.	Including motivation, job satisfaction, enjoyment, pride and so on.	
Patient treatment, communication, capacity and outcomes	Referral procedures/criteria, capacity and demand issues.	Including patient interventions and outcomes, and measurements of effectiveness.	Excluding communication and relationships with external services and organizations (4).
		Including throughput of patients, care-needs and issues of workload and time-management.	
		Including communication and relationships with patients and family members.	
Role mix, professional roles and responsibilities	Issues regarding the variety of roles and distribution of responsibilities currently within the team.	Including the balance between maintenance of professional roles and the need for generic working.	Excluding professional development (6) or service development activities (that is, developing/distributing skills and knowledge) (13).
		Excluding team size (11), team work issues (5).	
			Excluding lack of clarity of roles (5).
			Excluding functions ordinarily performed by external services (4).
Service development activities	Service development and team building activities.	Including case reviews and other reflective practices (for example, de-briefing procedures).	
		Including specific skill development across the team (for example, supporting changing roles).	
		Including group knowledge translation activities, for example, journal clubs and visits to other services.	

Table 4 demonstrates the triangulation of these three data sources to identify ten characteristics that emerged as underpinning a good interdisciplinary team. The high level of concordance across the three sources is illustrated. The only theme not explicitly identified from the thematic synthesis of the literature that was recognized by the teams was “clarity of vision”; however, this was partly covered by the literature review themes of values and professional commitment. It is interesting to note that an audit of intermediate care, published after this study was completed, recognized weaknesses in strategic planning by commissioners [37], which was highlighted as a reason for the lack of clear vision by team members in this study.

**Table 4:** Triangulation of the data sources to identify the characteristics of a good interdisciplinary team

**Data synthesis**	**Data sources**		
**Characteristics of a good interdisciplinary team**	**Themes from thematic synthesis of the literature**	**Themes identified as characteristics of a good team from IMT workshops**	**Topics identified by participants as challenges to interdisciplinary team work from IMT workshops**
Communication	Communication	Good communication	Communication and relationships-external
Individual characteristics	Individual characteristics	Personal qualities	
	Problem solving/decision-making		
	Interdependence		
Leadership and management	Leadership	Leadership and management	Management, leadership, decision-making and autonomy
Personal rewards, training and development opportunities	Learning	Training and development opportunities	Continuing professional development, rotation and career progression
		Individual rewards and opportunity	Morale and motivation
Quality and outcomes of care	Patient focus	Quality and outcomes of care	Patient treatment, communication, capacity and outcomes
Appropriate skill mix	Skills	Appropriate skill mix	Role mix, professional roles and responsibilities
	Team characteristics		
Appropriate process and resources	Structures	Appropriate team processes and resources	Facilities, resources, procedures and administration
Team climate	Climate	Team culture	Communication and relationships-internal
Respecting and understanding roles	Power	Respecting and understanding roles	Joint working
	Perceptions		Role mix, professional roles and responsibilities
	Roles		
Clarity of vision	Values	Clear vision	Clarity of vision, uncertainty and changes to service
	Professional commitment	External image of the service	
		Flexibility	

Not surprisingly, the team participants did not raise any challenges or issues related to individual characteristics. This illustrates the value of combining data anonymously reported in the literature with primary data from the teams themselves. While the interaction of individual characteristics is fundamental to the way the team functions, it is unlikely to be directly affected by team actions, with the exception of changing recruitment criteria. However, one of the outcomes of the larger IMT intervention undertaken as the main part of the study was that some teams perceived that they were able to develop individual competencies which better prepared them to work as a member of an interdisciplinary team. Further research is needed to understand the individual characteristics of an “interdisciplinary team member”.

The ten themes identified as the characteristics of a good interdisciplinary team are further described in the following Table 5.

**Table 5:** Characteristics of a good interdisciplinary team

**Themes**	**Description**
1. Leadership and management	Having a clear leader of the team, with clear direction and management; democratic; shared power; support/supervision; personal development aligned with line management; leader who acts and listens.
2. Communication	Individuals with communication skills; ensuring that there are appropriate systems to promote communication within the team.
3. Personal rewards, training and development	Learning; training and development; training and career development opportunities; incorporates individual rewards and opportunity, morale and motivation.
4. Appropriate resources and procedures	Structures (for example, team meetings, organizational factors, team members working from the same location). Ensuring that appropriate procedures are in place to uphold the vision of the service (for example, communication systems, appropriate referral criteria and so on).
5. Appropriate skill mix	Sufficient/appropriate skills, competencies, practitioner mix, balance of personalities; ability to make the most of other team members' backgrounds; having a full complement of staff, timely replacement/cover for empty or absent posts.
6. Climate	Team culture of trust, valuing contributions, nurturing consensus; need to create an interprofessional atmosphere.
7. Individual characteristics	Knowledge, experience, initiative, knowing strengths and weaknesses, listening skills, reflexive practice; desire to work on the same goals.
8. Clarity of vision	Having a clear set of values that drive the direction of the service and the care provided. Portraying a uniform and consistent external image.
9. Quality and outcomes of care	Patient-centered focus, outcomes and satisfaction, encouraging feedback, capturing and recording evidence of the effectiveness of care and using that as part of a feedback cycle to improve care.
10. Respecting and understanding roles	Sharing power, joint working, autonomy.

These characteristics can be re-formulated as competency statements that an effective interdisciplinary team functioning at a high level might be expected to demonstrate.

Competencies of an interdisciplinary team:

1. Identifies a leader who establishes a clear direction and vision for the team, while listening and providing support and supervision to the team members.

2. Incorporates a set of values that clearly provide direction for the team’s service provision; these values should be visible and consistently portrayed.

3. Demonstrates a team culture and interdisciplinary atmosphere of trust where contributions are valued and consensus is fostered.

4. Ensures appropriate processes and infrastructures are in place to uphold the vision of the service (for example, referral criteria, communications infrastructure).

5. Provides quality patient-focused services with documented outcomes; utilizes feedback to improve the quality of care.

6. Utilizes communication strategies that promote intra-team communication, collaborative decision-making and effective team processes.

7. Provides sufficient team staffing to integrate an appropriate mix of skills, competencies, and personalities to meet the needs of patients and enhance smooth functioning.

8. Facilitates recruitment of staff who demonstrate interdisciplinary competencies including team functioning, collaborative leadership, communication, and sufficient professional knowledge and experience.

9. Promotes role interdependence while respecting individual roles and autonomy.

10. Facilitates personal development through appropriate training, rewards, recognition, and opportunities for career development.

In addition, our study identified the need for teams to regularly invest time in the processes of team development and maintenance of team functioning to ensure that these competencies are entrenched and enacted in their daily practice. Recognition of such time is frequently omitted from randomized controlled trial evidence of interprofessional working and is correspondingly overlooked when performing cost effectiveness evaluations.

## Discussion

### Limitations of the approach

The systematic review, which sought to identify quantitative studies detailing the outcomes of different staffing models, proved most amenable to conventional methods of systematic review and did not require significant amendment from its original protocol. However, the review team encountered the now-familiar deficit in contextual richness or “thickness” within the quantitative studies and had to compensate with strategies specifically seeking qualitative research studies or process evaluations [38]. We acknowledge, in association with guidance provided by the Cochrane Collaboration Qualitative Methods Group, that qualitative evidence from studies from the same source as the trials (within study reports or “sibling studies”) would reduce the contextual variation between different configurations of interdisciplinary working. However given the acknowledged absence of such studies [34], the best available solution was to bring together the two evidence bases and then to triangulate them with rich primary qualitative research data.

This study has not examined the interaction between the characteristics of interdisciplinary teams and it is possible that there is some interdependence between some of the characteristics. For instance, previous research suggests that good leadership may be required for the team to have strong clarity of vision [39]. Further exploration and validation is required to examine whether any causal relationships exist between the different components of interdisciplinary team work.

The facilitation process used in the workshops in this study was informed by the available literature, and therefore has the potential to bias the results from the teams. The risk of researcher bias was lessened by having the teams facilitated by six different facilitators. It is notable that the views of the teams, and the issues they faced, were all similar. The high level of concordance between the published literature and the findings from the teams suggests strong face validity for the characteristics described and competencies proposed in this paper.

The results presented in this paper are derived from interdisciplinary teams involved in the delivery of CRAICs. As such, they involve a specific, but broad, range of disciplines. Previous literature has shown that these groups are typified by being non-medically led, non-hierarchical and fairly democratic in their approaches [24]. This research, and previous research [30,31], also found a great deal of heterogeneity in the structure and organization of these teams. It is therefore not possible to assume that these findings are relevant to all interdisciplinary teams. Further research will be required to examine the generalizability of these characteristics and competencies beyond this paper.

By establishing a broad set of competencies to guide interdisciplinary team work it moves towards the identification of a suite of processes to which teams can adhere, and sets up mechanisms and areas for improvement. As the published literature demonstrates, few existing interventions around interdisciplinary team work focus on these competencies and processes to implement them. Instead such studies tend to examine a single mechanism to support interdisciplinary team work. The characteristics and competencies identified in this study provide a framework for investigating good interdisciplinary team work, how it might be examined in different contexts, and how teams might identify interventions to improve or optimize their interdisciplinary team work.

## Conclusions

Interdisciplinary health care teams face a set of challenges that are not necessarily encountered by other types of teams such as unidisciplinary or non-health care teams. These challenges include the contentious nature of sharing professional roles and expertise, planning and decision-making, while delivering quality patient care within complex contexts. This paper combines quantitative and qualitative insights from the published literature with empirical data derived from the experiences and insights of interdisciplinary teams working in CRAICs to identify the characteristics of a good interdisciplinary team. Our research has drawn together these sources of evidence to elicit a theoretical understanding and develop a framework to define the characteristics of interdisciplinary team work and presented these as competencies for effective interdisciplinary team work. These outcomes now need to be validated with other types of interdisciplinary teams to determine their level of transferability to other teams and contexts.

## Abbreviations

CRAICs: Community rehabilitation and intermediate care services; IMT: Interdisciplinary Management Tool; UK: United Kingdom.

## Competing interests

The authors declare they have no competing interests.

## Authors’ contributions

SN, PE and AS conceived the original project and obtained the funding, were involved in the data collection, analysis, preparation of the final report and drafting and amending the final manuscript. SA was involved in all stages of the project implementation, data collection and analysis, drafting the final report, and contributed to this manuscript. AR performed further analysis, drafting and compilation of the final report. All authors read and approved the final manuscript.

## Disclaimer

Department of Health disclaimer: “The views and opinions expressed therein are those of the authors and do not necessarily reflect those of the HS&DR program, NIHR, NHS or the Department of Health”.

Target journal: Human Resources for Health
